# Supplementary material for: RNA-encoded PGT121-LS anti-HIV antibody: Comprehensive preclinical characterization and translational pharmacokinetics
Source: Mol Ther Nucleic Acids. 2026 Sep 17;37(4):103102. doi: 10.1016/j.omtn.2026.103102 (PMC13676711; doi:10.1016/j.omtn.2026.103102)
Supplement: Supplementary file 1 [file mmc1.pdf]

OMTN, Volume *37*

Supplemental information

RNA-encoded PGT121-LS anti-HIV antibody: Comprehensive preclinical characterization and translational pharmacokinetics

Felix Tolksdorf, Johannes Nelke, Robin Johannson, Janina Caesar, Anuhar Chaturvedi, Anja Kopp, Leyla Fischer, Alexandra Malz, Sven Kratochvil, Imke Gerhard, Jan P. Bogen, Candice Morin, Maximilian Kullmann, Michael S. Seaman, Georgia D. Tomaras, Nicole L. Yates, Margaret E. Ackerman, Joshua A. Weiner, Ursula Ellinghaus, Christiane R. Stadler, Uğur Şahin, and Valentin Le Douce

Supplemental Tables

Table S1 Physicochemical parameters of LNP batches

Experiment	RNA-encoded construct[1]	Encapsulated RNA (mg/mL)	Encap-sulation (%)	Yield (mg)	RNA/lipid ratio (mg/µmoL)	Particle size (nm)	Poly-dispersity
Figure 1 C, E Figure 3 A, B	PGT121	1.00	87%	3.28	0.023	78	0.068
Figure 1 C, E Figure 3 A, B	PGT121-LS	1.00	88%	3.32	0.024	77	0.061
Figure 1 C, E Figure 3 A, B	PGT121-del294	1.00	87%	3.28	0.023	77	0.072
Figure 1 C, E Figure 3 A, B	PGT121-del294-LS	1.00	87%	3.06	0.023	79	0.570
Figure 4	PGT121-LS	2.85	91%	175	0.023	53	0.094

1 Each LNP contains RNA encoding heavy chain and light chain in a 1.5:1 HC:LC ratio

Table S2 Summary of hematology

Table S3: Summary of clinical chemistry

Table S4: Summary of coagulation

Table S5: Allometric exponents (b) for Fc-engineered mAbs79 and RibobNAbs, as well as NHP and human PK parameters

Parameter	Exponent NHP	NHP parameters	Predicted human parameters
V_c	0.95	2.32 (L)	2.03 (L)
V_p	0.95	1.77 (L)	1.55 (L)
CL	0.55	0.13 (L/day)	0.04 (L/day)
Q	0.6	0.74 (L/day)	0.25 (L/day)
k_a*	1		
F*	1		

Abbreviations: Fc = fragment crystallizable; mAb = monoclonal antibody; NHP = non-human primate; V_c = volume of distribution in the central compartment; V_p = volume of distribution in the peripheral compartment; CL = clearance from the central compartment; Q = intercompartmental clearance between central and peripheral compartment, *RibobNAb specific parameters: k_a= translational rate, F= translational efficiency.

Supplemental Figures

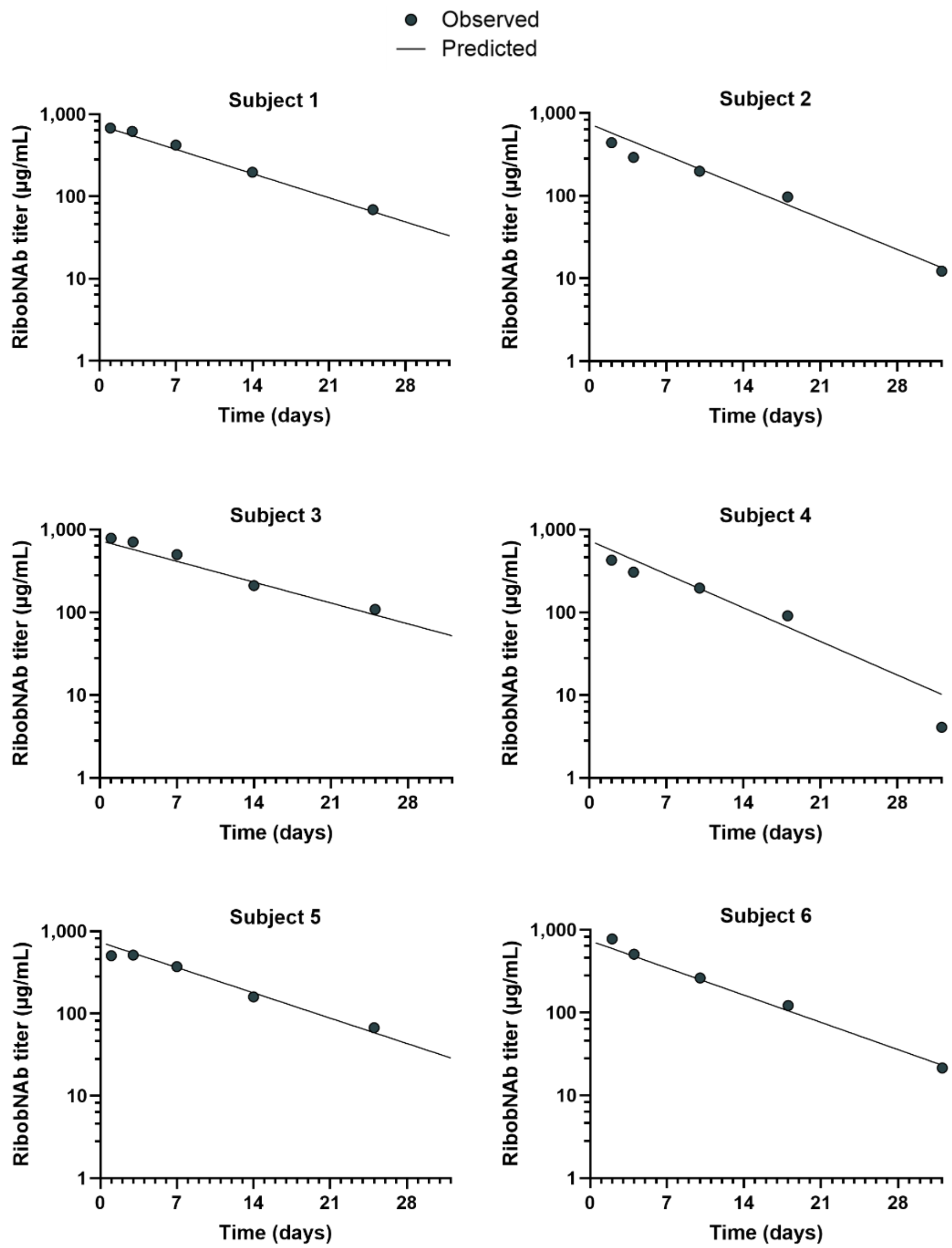

Figure S1: Individual profiles of observed and predicted PGT121 RibobNAb concentrations versus time after a single IV dose in NSG Tg32 mice

Male and female NSG Tg32 mice (n = 6 per group) expressing hFcRn received a single IV bolus of 30 μg RNA-LNPs encoding PGT121 RibobNAb. Blood samples were collected at the indicated time points, and RibobNAb serum concentrations were determined using ELISA. Observed data are shown as symbols. Predicted RibobNAb serum concentrations from the established population PK model are shown as solid lines.

hFcRn = human neonatal Fc receptor; IV = intravenous; NSG Tg32 = NOD scid gamma mice expressing hFcRn; RibobNAb = RNA-encoded, LNP-formulated anti-HIV-1 broadly neutralizing antibody.

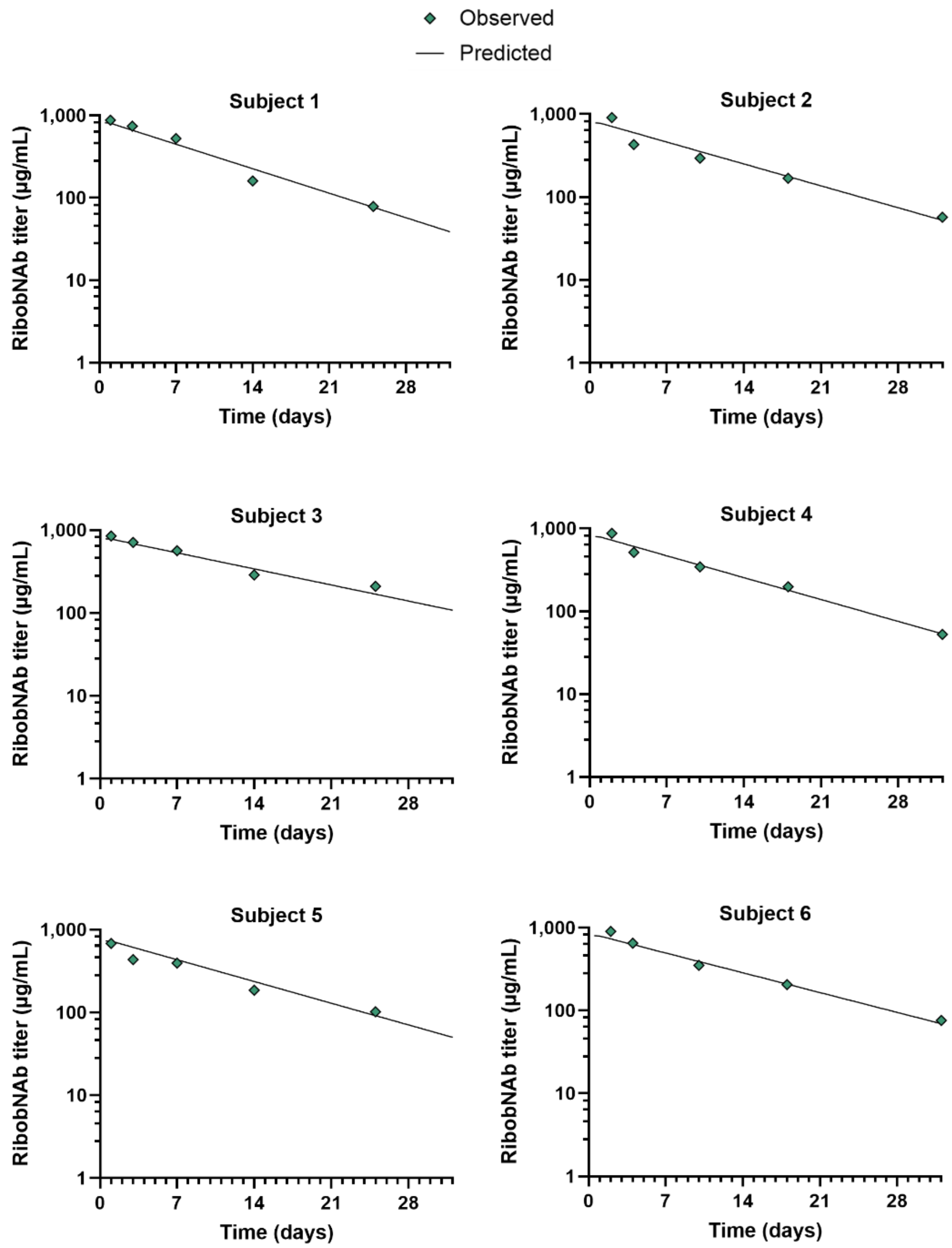

Figure S2: Individual profiles of observed and predicted PGT121-LS RibobNAb concentrations versus time after a single IV dose in NSG Tg32 mice

Male and female NSG Tg32 mice (n = 6 per group) expressing hFcRn received a single IV bolus of 30 µg RNA-LNPs encoding PGT121-LS RibobNAb. Blood samples were collected at the indicated time points, and RibobNAb serum concentrations were determined using ELISA. Observed data are shown as symbols. Predicted RibobNAb serum concentrations from the established population PK model are shown as solid lines.

hFcRn = human neonatal Fc receptor; IV = intravenous; NSG Tg32 = NOD scid gamma mice expressing hFcRn; RibobNAb = RNA-encoded, LNP-formulated anti-HIV-1 broadly neutralizing antibody.

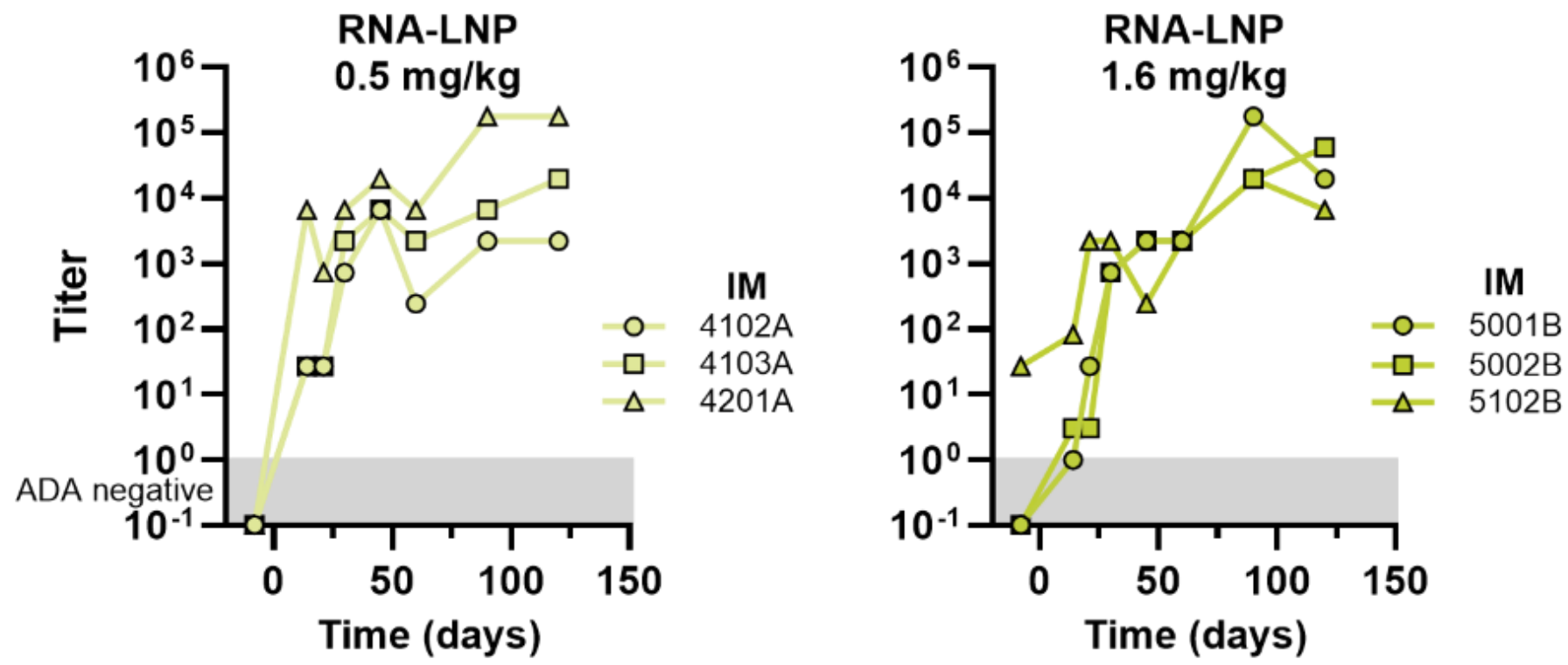

Figure S3: ADA titers in NHP IM groups

ADA positive samples were further titrated by 3-fold dilution until no significant difference was observed, with the last significant dilution step determining the ADA titer. Data for individual animals are shown.

ADA = anti-drug antibody; IM = intramuscular; LNP = lipid nanoparticle;RNA = ribonucleic acid.

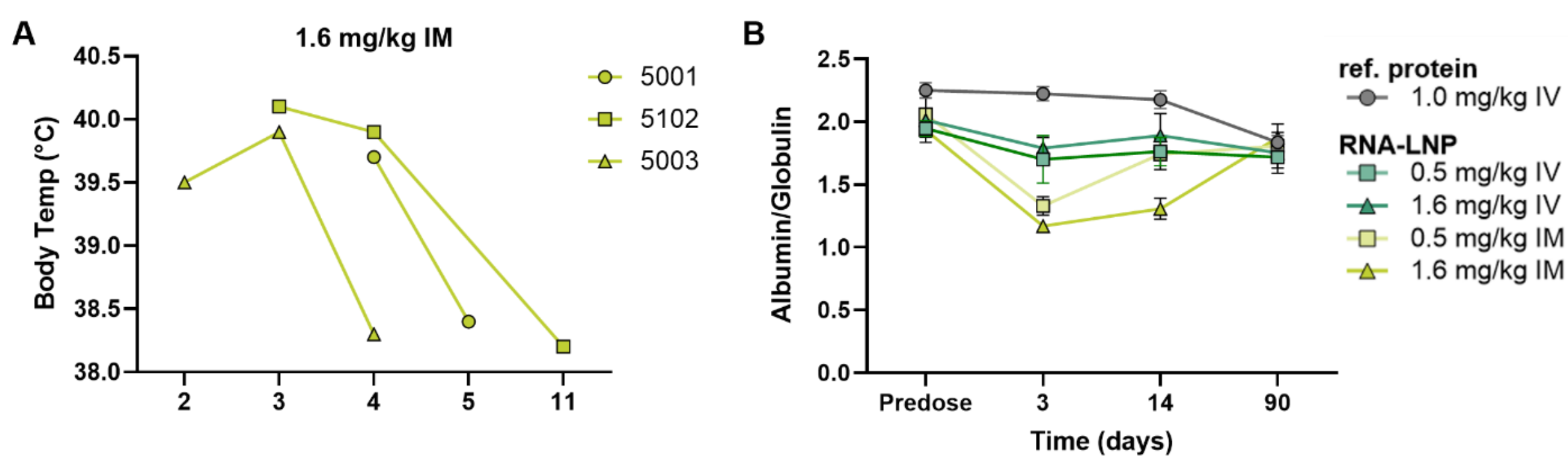

Figure S4: Body temperatures and albumin/globulin ratios in NHPs

A) Hyperthermia was noted in animals treated with 1.6 mg/kg PGT121-LS RNA-LNPs (animal nos. 5001, 5102 and 5003) from Day 2 to Day 4 with a return to normal body temperatures thereafter. B) Albumin/globulin ratios were determined predose and on Days 3, 14 and 90. IM injection of PGT121-LS RNA-LNPs resulted in a lower albumin/globulin ratio on Days 3 and 14.

IM = intramuscular; IV = intravenous; LNP = lipid nanoparticle; ref. = reference; RibobNAb = RNA-encoded, LNP-formulated anti-HIV-1 broadly neutralizing antibody; RNA = ribonucleic acid; Temp = temperature.
